# Internet-based Self-Assessment after the Tsunami: lessons learned

**DOI:** 10.1186/1471-2458-11-18

**Published:** 2011-01-07

**Authors:** Stefan Vetter, Astrid Rossegger, Thomas Elbert, Juliane Gerth, Frank Urbaniok, Arja Laubacher, Wulf Rossler, Jérôme Endrass

**Affiliations:** 1 Centre for Disaster and Military Psychiatry, University of Zurich, Militaerstrasse 8, 8021 Zurich, Switzerland; 2 Psychiatric/Psychological Service, Department of Justice, Canton of Zurich, Feldstrasse 42, 8004 Zurich, Switzerland; 3 University of Konstanz, 78457 Konstanz, Germany; 4 Research Unit for Clinical and Social Psychiatry, Psychiatric University Hospital, Zurich, Lenggstrasse 31, 8032 Zurich, Switzerland

## Abstract

**Background:**

In the aftermath of the Tsunami disaster in 2004, an online psychological self-assessment (ONSET) was developed and made available by the University of Zurich in order to provide an online screening instrument for Tsunami victims to test if they were traumatized and in need of mental health care. The objective of the study was to report the lessons learnt that were made using an Internet-based, self-screening instrument after a large-scale disaster and to discuss its outreach and usefulness.

**Methods:**

Users of the online self-assessment decided after finishing the procedure whether their dataset could be used for quality control and scientific evaluation Their answers were stored anonymously only if they consented (which was the case in 88% of the sample), stratified analyses according to level of exposure were conducted.

**Results:**

A total of 2,914 adult users gave their consent for analysis of the screenings. Almost three quarter of the sample filled out the ONSET questionnaire within the first four weeks. Forty-one percent of the users reported direct exposure to the Tsunami disaster. Users who were injured by the Tsunami and users who reported dead or injured family members showed the highest degree of PTSD symptoms.

**Conclusion:**

ONSET was used by a large number of subjects who thought to be affected by the catastrophe in order to help them decide if they needed to see a mental health professional. Furthermore, men more frequently accessed the instrument than women, indicating that Internet-based testing facilitates reaching out to a different group of people than "ordinary" public mental health strategies.

## Background

### Prevalence of PTSD after Natural disasters

According to Galea, Nandi, and Vlahov [1], the prevalence of Post-Traumatic-Stress Disorder (PTSD) is generally lower after natural disasters (e.g. earthquakes, hurricanes, floods) than after human-made/technological disasters. In the latter, there is usually an obvious group of direct victims, whereas in the former, it is more difficult to identify who was affected and to what degree and thus to assess the impact on public mental health. Depending on the definition of what constitutes the affected population, the literature reports prevalence rates of Post-Traumatic-Stress Disorder (PTSD) of 5 up to 60 percent - though the majority of the research papers report prevalence rates of less than 30 percent [1].

Traumatized individuals benefit from brief treatment programs, especially if their symptoms do not become chronic [2-4]. From a public mental health point of view it is thus important to offer easy and unbureaucratic access to assistance and interventions that provide mental health care to disaster victims [5]. According to the British NICE clinical guideline it is recommended to use a brief screening instrument for PTSD routinely one month post-trauma [6]. In smaller populations affected by a disaster, telephone monitoring seems to be a viable approach for coming into contact with PTSD patients in order to encourage them to seek out treatment [7]. However, a routine screening could easily be stretched to its limits in terms of expert resources, costs and effort. A unique way to reach out and monitor is made available through the Internet. So far, Internet-based mental health applications have mainly been used to distribute information or to collect it for research purposes but were not yet employed as screening instruments after large-scale disasters [8]. The principal advantage of an Internet-based screening is the outreach and the cost-efficiency of the instrument. In countries affected by a natural disaster, where the infrastructure - namely IT networks, distribution of electricity, etc. - remained largely functional or were reestablished within a month after the disaster, using an Internet-based instrument can facilitate the screening process. There are, however, several limitations to an Internet-based instrument. Since this technology is quite new, knowledge on the usefulness of digital questionnaires after disasters is very limited. It is unclear, whether those individuals who are affected, will accept this form of contacting by the authorities. Using common sense, it can be assumed, that the elderly will be reluctant using such an instrument and that those individuals who do not have regular access to the Internet will have even more difficulties to gain access in a post-disaster scenario. It can be furthermore assumed, that those individuals who do not have regular access are more likely to be on the fringe of society, more likely to be economically marginalized and thus are at higher risk to suffer from various stressors. Hence, Internet-based questionnaires can very well help to reach out to a considerable proportion of the community hit by a disaster but in fact miss the most disadvantaged and thus those most severely hit by the catastrophe. A further disadvantage of an Internet-based questionnaire is obviously that it is highly standardized and that it is not possible to assist participants who do not understand certain parts of the instrument. Furthermore, the result of the screening relies fully on self-assessment and Internet-based questionnaires are likely to inflate scores [9] leading partially to false positive or over-diagnosed individuals. On the other hand, there is evidence that psychometric properties of Internet-based questionnaires are not biased and that questionnaire format and presentation order do not affect rates of psychological symptoms reported by participants [10]. There is evidence, that there are no significant differences between assessment techniques [11,12], suggesting that Internet-based methods are a suitable alternative to more traditional methods.

The objective of this article was to report the experiences that were made using an Internet-based, self-screening instrument after a large-scale disaster. Furthermore we wanted to discuss the outreach and usefulness of the instrument.

## Methods

### Setting

Newspaper articles published in the time period after the Tsunami disaster on December 26^th ^2004, reported approximately 230,000 deaths and 110,000 missing people worldwide, including 2,000 to 3,000 possibly affected Swiss tourists. More than 3,800 declarations of missing persons were filed at the Swiss Federal Department of Foreign Affairs, and Swiss Tsunami losses added up to 110 casualties and 100 severely injured persons [13]. The Swiss Federal Office for Civil Protection concluded that there were not enough trained professionals available who could personally contact all persons who claimed to be affected by the disaster and thus supported the development of an easily accessible screening instrument. As a consequence of the widespread use of Internet applications in Switzerland, the Centre for Disaster and Military Psychiatry at the University of Zurich decided to develop an Internet-based self-screening instrument for post-Tsunami stress reactions. The instrument was developed with the intention of providing Tsunami survivors with unbureaucratic and readily accessible support, by helping the affected population to assess their level of traumatization and to assess the need of seeing a mental health professional. Since ONSET was developed as a response to the Tsunami-wave and had to be translated into French, Italian, and English, it was not possible to go online four weeks after the event as recommended by the NICE-guideline [6] but rather six weeks after the disaster. ONSET was available on a special homepage of the University of Zurich between February 18^th ^and August 31^st ^2005. Due to an extensive and free introduction campaign in the Swiss media (newspaper, journals, radio and television) along with further information distributed by health insurance companies, as well as the Department of Foreign Affairs (also see Table 1), ONSET rapidly gained a high level of awareness in the Swiss public and has raised interest outside of Switzerland.

**Table 1:** Summary of the public relation activities to announce ONSET

Point in time	Public relation effort
01/24/2005-01/30/2005	Announcement of the soon available ONSET on the websites of the 10 largest Swiss health insurance providers. These companies contacted by letter and email all their insurants, who were known to have been affected by the Tsunami.

02/01/2005-02/28/2005	Swiss Department of Foreign Affaires informed all Swiss citizens living abroad via email about the availability of ONSET.

02/14/2005	1^st ^press release of the public relation department of the University of Zurich (Unipublic) about the upcoming availability of ONSET, covering the entire press as well as electronic media.

02/18/2005	Information about ONSET by the Swiss Federal Coordinated Medical Services to all their partners.

02/18/2005-08/31/2005	Telephone hotline for ONSET users, general practitioners and mental health professionals by the Centre for Disaster and Military Psychiatry (CDMP). For the same target audience contact by email was also possible.

02/18/2005-04/30/2005	Hundreds of press, radio and TV interviews given by co-workers of CDMP concerning ONSET; making it almost impossible to sustain normal work flow.

02/20/2005-02/28/2005	Transmission of a press release about ONSET to the major press houses, radio and TV station in the neighbour countries Germany, Austria, Italy and France by the CDMP.

04/01/2005	2^nd ^press release of Unipublic (University of Zurich) of ONSET use and some preliminary findings.

12/31/2005	ONSET's public relation campaign was the most successful activity of Unipublic (University of Zurich) with several hundreds citations, articles, broadcasts and electronic media in 2005.

### ONSET

The general layout of ONSET followed modern principles of Internet-based psychological testing and assessment [14-16]. The entire screening contained 65 questions, presented on 6 screens. It took about 12 minutes to answer them. In order to use ONSET, the user had to complete a registration first. Users could choose their own logins and passwords. This login procedure allowed the users to fill out ONSET several times per person. Never during registration and assessment were any email addresses or other personal data gathered, making a completely anonymous test use possible. Due to further requirements of Swiss data privacy protection, IP addresses were likewise not recorded.

In order to cover a large part of the population, three different subtests of ONSET for different age groups (adults [17 years and older], adolescents [11-16 years], and children [4-10 years]), each in four different languages (German, French, Italian, and English) were made available.

Demographic information was restricted to gender, age, origin, country of residence, and education in order to guarantee strict anonymity. Thereafter, users were asked to answer the following seven questions concerning their exposure to the disaster: "*Did you witness the Tsunami wave yourself?", "Were you hurt?", "Did you lose any family members (parents, siblings, children, partners etc.)?", "Did any family members get hurt and/or are they in hospital in the crisis area or in Switzerland?", "Did you lose any friends and/or acquaintances?", "Were any of your friends and/or acquaintances hurt and/or are they in hospital in the crisis area or in Switzerland?", "Did you lose any possessions as a consequence of the Tsunami earthquake in Asia?"*. Pre-existing traumatization was not assessed in order to keep the screening as short as possible. The severity of PTSD symptoms was assessed using the adapted Posttraumatic Stress Scale 10 (PTSS-10) [17,18], which screens with 10 questions like the following: *"I suffer from....sleep problems, ....nightmares, .....depression, .... fear of places and situations, which remind me of the events"*. The PTSS-10 was developed as a clinical screening instrument to identify people at risk of developing post-traumatic stress reactions (as defined in the DSM-III-R) and has good face validity. Due to its advantage as a brief screening inventory for PTSD "caseness", it was frequently used in recent, especially Scandinavian disaster research [17,18]. With the help of the PTSS, PTSD symptoms not necessarily related to the Tsunami disaster were assessed.

In order to also assess symptoms of potential comorbid disorders such as depression, anxiety, and obsessive-compulsive disorder, items covering the diagnostic criteria of these disorders according to DSM-IV were screened as well [19]. Due to copyright issues and the difficulty of the multi-lingual design of the instrument - besides the PTSS - no validated instruments could be used.

After completing the test users obtained a screening result listing their answers to the questions and one of three possible statements which either indicated that there was no need to worry about one's clinical state, or that one showed somewhat elevated levels of psychological stress or that one showed severe level of psychological strain and one was advised to contact a psychologist or a physician. This procedure was in line with the NICE guideline where it is recommended that screened individuals, who are at risk for mental disorder, are advised to contact their general health practitioners for further assessment. Additionally, in order to facilitate the contacting of general health practitioners, users were able to print out their screening results to bring along to their doctor's appointment.

Recommendations were given depending on the acquired scores of the different assessment scales that were included in ONSET. Seeing a physician or mental health professional was recommended if ONSET revealed a suicidal ideation or high scores in any of the following scales: PTSS, depression-scale, the items addressing anxiety disorder, obsessive compulsive disorder. The PTTS-10 the cut-off was raised to 14 (compared to 12 defined in the PTTS-10 manual), since it has been shown that self-evaluations via Internet might lead to up to 10-20% higher scores [9]. Within the 10-item depression-scale we set the cut-off at ≥ 9. For all dichotomous items one point was allocated for each positive answer, except from the one item on suicidal thoughts that by itself scored 10 points. Concerning Anxiety Disorder (12 items) and Obsessive Compulsive Disorder (13 Items) the need for consulting a doctor was set to the upper 30% of the possible scoring range. The subscales proved to be internally consistent: PTSS, CR-alpha = 0.87; Depression items, CR-alpha = 0.82; Anxiety items, CR-alpha = 0.75; OCD items, CR-alpha = 0.78.

### Using ONSET for research proposal

After the screening, users were invited to give informed consent for their test data to be used anonymously for scientific analysis. Consent was confirmed by typing-in an alphanumeric code, which was newly generated for each person by the software. If they denied consent, their data were not stored but immediately deleted. If they agreed, their results, the date and time of assessment as well as the logins were saved. The login was used until the end of the above-mentioned assessment period so that users could, by email or letter, ask to have their data deleted. This login procedure led also to the problem, that with one login it was possible to fill out ONSET several times per person.

### Ethical approval

The study was approved by an external ethics committee, the Zurich Cantonal Ethics Committee. ONSET was classified by the ethics committee as 1) a completive mental healthcare service, which 2) guaranteed full anonymity and 3) used only the data of those users who gave their consent for their data to be used for scientific evaluation of ONSET.

### Study population

During the six months ONSET was available online, 4,161 registered adult users (17 years or older) from 61 nations and 5 continents participated, which yielded 3,313 complete adult data sets. Furthermore there were only a very small number of children (N = 23) and adolescents (16 years or younger) (N = 87) filling out the age specific subversions of the questionnaire.

Due to the small sample size of children and adolescents the analyses were restricted to adults only. 2,921 (88%) of the adult users gave their informed consent for the analysis of the screenings. 7 ONSET users who declared being older than 85 years old were excluded from further analyses due to inconsistencies in their answers suggesting a bogus self-report. This led to a final sample size of N = 2'914. Of the 2914 responders, 2895 logged in and responded within one calendar day. It is possible that the 19 users, whose login and response date diverged, filled out the questionnaires more than once.

### Statistical Analysis

The first step in the analysis strategy aimed at identifying the characteristics of the study sample using descriptive statistics. In a further step, we decided to investigate whether the composition of the ONSET sample varied over time. The first four weeks were collapsed into one category (phase 1), week five to eight into the second category (phase 2), and for the remaining time period (nine weeks until six month) we created a third category (phase 3). We then analyzed the characteristics of the study sample across the time-strata (phase 1 to 3).

For a better understanding of the ONSET sample, the total sample was divided into five subgroups according to their degree of exposure, group 1 including individual with highest level of exposure and group 5 the lowest level of exposure. If an ONSET user reported having suffered from various degrees of exposure, the user was allocated to a group according to the highest level of exposure. Stratified statistical analyses were performed for these five groups. In order to better understand and compare the degree of traumatic stress symptoms in people with different types of exposure, the psychiatric symptom scores were T-transformed (mean = 50, standard deviation = 10).

The relationship between the level of exposure and psychiatric symptoms and between level of exposure and time of participation was analyzed using ANOVA with posthoc Scheffée tests. The associations between suicidal ideation, re-experience of the traumatic event and exposure to the Tsunami were assessed using bivariate logistic regression analysis. In these logistic models suicidal ideation and re-experience served as dichotomous dependent variables and exposure to the event was defined as a dummy-coded predictor variable. The data was analyzed using STATA SE 10.0 [20].

## Results

### Socio-demographic characteristics of ONSET-users (N = 2'914)

More than three-quarter of the users (76.2%, n = 2219) were Swiss citizens and 81.1% (n = 2364) Swiss citizens or Swiss residents.

In total 77.8% (n = 2267) of the screened persons reported living in Switzerland, 9.2% (n = 268) living in Italy and 6.5% (n = 190) living in Germany.

Among the sample, the mean age was 37.5 years (SD = 13.1 yrs; range: 17-85 yrs.). 57.5% (n = 1674) of the users were male and 42.5% (n = 1240) were female. 53.5% (n = 1559) of the users were single, 34.8% (n = 1013) were married, 9.7% (n = 282) were divorced and 2.1% (n = 60) were widowed. 32.3% (n = 942) of the persons screened specified vocational training as their as highest educational degree, 16.4% (n = 478) a degree from a university of applied sciences and 22.5% (n = 655) a university degree. 15.0% (n = 436) of persons screened were currently attending secondary school, while 6.7% (n = 195) specified compulsory school attendance as their highest educational degree. 7.1% (n = 208) could not be categorized in any of these groups.

### Exposure

Forty-one percent (N = 1207) of the users were affected by the Tsunami disaster in that they reported direct experience and in some cases even injuries resulting from the disaster, as well as injured or lost relatives. Accordingly, six out of ten ONSET users (58.6%, N = 1707) were not directly affected by the disaster. 467 persons (19.8%) being Swiss or living in Switzerland witnessed the Tsunami themselves. More detailed information about the prevalence of the different types of exposure for the whole sample as well as for Swiss citizen and/or Swiss residents is displayed in Table 2.

**Table 2:** Prevalence of types of exposure reported by the participants (multiple answers were possible)

	All (n = 2914)	Swiss Citizen/Swiss Residence
	**N (%)**	**N (%)**

Injured friends/acquaintance	570 (19.6)	486 (20.6)

Witnessed the tsunami themselves	565 (19.4)	467 (19.8)

Lost friends/acquaintance	497 (17.1)	398 (16.8)

Lost property	324 (11.1)	257 (10.9)

Were injured	163 (5.6)	118 (5.0)

Lost family member	151 (5.2)	111 (4.7)

Injured family member	144 (4.9)	114 (4.82)

No exposure	1'707 (58.6)	1'364 (57.7)

Analyzing the five level of exposure subgroups, 5.6% (N = 163) of group 1 reported to be have been physically injured during the Tsunami events, 14.1% (N = 412) of group 2 witnessed the Tsunami-wave themselves, 4.8% (n = 141) of group 3 reported injured or dead family members, 16.9% (N = 491) of group 4 and 58.6% (N = 1707) of group 5 stated that they were not exposed to the Tsunami and could thus be interpreted as a control group (see Table 3).

**Table 3:** Severest form of exposure of each participant and recommendation to seek help

		Exposure	Recommendation to seek help
		**N (%)**	**N (%)**

Group 1	Were injured	163 (5.6)	115 (70.6)

Group 2	Witnessed the Tsunami	412 (14.1)	268 (65.1)

Group 3	Injured or dead family members	141 (4.8)	104 (73.8)

Group 4	Injured or dead friends/acquaintance, lost property	491 (16.9)	310 (63.1)

Group 5	No exposure	1,707 (58.6)	795 (46.6)

	*Total*	*2,914 (100)*	*1952 (54.6)*

### ONSET-stratified by time periods

72.5% (N = 2112) of the ONSET-users responded within the first month (phase 1), 16.2% (472) within the second month (phase 2) and 11.3% (330) in the third phase until the instrument went offline. Almost three quarter of the sample filled out the ONSET questionnaire within the first four weeks. Participation steadily declined with only 13 persons registering in the last two weeks to participate.

When comparing the composition of the strata across the three phases, it becomes evident, that ONSET users in Phase 2 were more likely to be directed affected by the Tsunami-wave. However, in absolute figures, there were still more users, who witnessed the wave in Phase 1 than in Phase 2 (n = 351 vs. n = 143).

Table 4 gives an overview over the characteristics of onset users stratified for the three phases.

**Table 4:** Characteristics of ONSET-users stratified by three time-phases

	Phase 1 % (n)	Phase 2 % (n)	Phase 3 % (n)
Gender: Male	57.8 (1224)	51.0 (241)	65.2 (215)

Age (mean)	37.7	39.9	32.5

Swiss citizen	78.9 (1670)	65.8 (311)	73.0 (241)

Witnessed the Tsunami themselves	16.6 (351)	30.3 (143)	21.5 (71)

Were injured themselves	4.6 (96)	9.3 (44)	7.0 (23)

Lost family member	4.6 (96)	6.1 (29)	7.8 (25)

Injured family member	4.5 (94)	6.1 (29)	6.4 (21)

Lost friends/acquaintance	15.4 (326)	24.2 (114)	17.3 (57)

Injured friends/acquaintance	18.7 (394)	24.8 (117)	17.9 (59)

Lost property	9.7 (205)	16.5 (78)	12.4 (41)

### Examined mental health symptoms

#### Traumatic alarm reaction (physiological state of alarm as a consequence of imminent threat or danger)

Among the 2'914 users, the mean score of the PTSS-10 was 12 points (SD = 7.1). Using a cut-off of 12 points, 45.0% (n = 1,308) of users reported a degree of symptomatology relevant to PTSD. An adjusted cut-off of 14 points reduced the prevalence rate of respondents showing symptoms relevant to PTSD by almost 10 percent to 35.7% (n = 1037). In group 1 (injured by the Tsunami) 53.1% (n = 86) showed symptoms relevant to PTSD with a PTSS-10 score of 14 or higher, in group 2 (witnessed the Tsunami) it was 47.1% (n = 194), in group 3 (injured or dead family members) 54.6% (n = 77), in group 4 (injured or dead friends, lost property) 42.7% (n = 209) and in the unaffected group 5 27.7% (n = 471) showed symptoms relevant to PTSD.

After T transformation (M = 50; SD = 10) of the raw scores of the psychiatric symptoms and stratification for the five groups, users that were injured by the Tsunami themselves (group 1), and users that reported dead or injured family members (group 3) showed the highest degree of PTSD symptoms (M = 54.9, SD = 8.8). Their symptoms were significantly higher than those of users who reported injured or dead friends/acquaintance, who lost property (group 4, M = 51.3, SD = 9.4) or who did not report any direct form of exposure (group 5, M = 48.0, SD = 9.7). Users of this group reported significantly lower scores on the PTSS than all other groups.

#### Depression

Overall, the five groups differed significantly with respect to their degree of depressive symptoms. In detail, users in group 3 (dead or injured family member) showed the highest level of depressive symptoms (M = 55.1, SD = 9.2) followed by those users who were injured by the Tsunami (group 1, M = 53.7, SD = 10.3). Users reporting no exposure (M = 48.2, SD = 9.8) showed significantly lower scores on the PTSS than all other groups.

#### Anxiety

Overall, the five groups differed significantly in respect to their degree of anxiety symptoms. The highest symptoms of anxiety were found in the group of victims injured by the Tsunami (group 1, SD = 55.7, SD = 11.6), followed by those who reported injured or dead family members (M = 53.4, SD = 9.8). The lowest levels of symptoms of anxiety was reported by those who did not suffer from any direct impact of the disaster (group 5, M = 48.2, SD = 9.5).

#### Obsessive-Compulsive

Overall, the five groups differed significantly with respect to their degree of obsessive-compulsive symptoms. The highest levels were reported by group 1 (M = 52.2, SD = 11.8) followed by those ONSET users who reported injured or dead family members. The study participants who declared that they had injured or dead friends or had lost property in the disaster displayed slightly lower scores (M = 52.1, M_group3 _= 9.4, M_group4 _= 10.4). Users with no direct exposure reported the lowest levels of obsessive-compulsive symptoms (Group 5, M = 49.1, SD = 9.6).

Table 5 gives an overview over T-transformed mean scores in of the five groups as well as the ANOVA tables.

**Table 5:** Posttraumatic stress and comorbid symptomatology - on a T-transformed scale - stratified into the five different exposure groups

	Mean (SD)	Mean (SD)	Mean (SD)	Mean (SD)	Mean (SD)	Mean (SD)	ANOVA	PH	PH	PH	PH	PH	PH	PH	PH	PH	PH
	All	G 1	G 2	G 3	G 4	G 5		1 vs 2	1 vs 3	1 vs 4	1 vs 5	2 vs 3	2 vs 4	2 vs 5	3 vs 4	3 vs 5	4 vs 5

PtSS	50.0	54.9	53.3	54.9	51.3	48.0	p < 0.001^1^	ns	ns	p < 0.05	p < 0.01	ns	ns	p < 0.01	p < 0.01	p < 0.01	p < 0.01
												
	10.0	10.4	9.7	8.8	9.4	9.7											

Depression	50.0	53.7	52.0	55.1	51.9	48.2	p < 0.001^2^	ns	ns	ns	p < 0.01	p < 0.05	ns	p < 0.01	p < 0.05	p < 0.01	p < 0.01
												
	10.0	10.3	9.5	9.2	9.7	9.8											

Anxiety	50.0	55.7	52.6	53.4	51.1	48.2	p < 0.001^3^	p < 0.05	ns	p < 0.01	p < 0.01	ns	ns	p < 0.01	ns	p < 0.01	p < 0.01
												
	10.0	11.6	10.0	9.8	9.5	9.5											

Obsessive-Compulsive	50.0	52.2	49.7	52.1	52.1	49.1	p < 0.001^4^	ns	ns	ns	p < 0.01	ns	p < 0.05	ns	ns	p < 0.05	p < 0.01
												
	10.0	11.8	9.8	9.4	10.4	9.6											

Legend: ^1 ^= df (4/2899), F (53.04), N (2904), ^2 ^= df (4/2909), F (38.42), N (2914), ^3 ^= df (4/2909), F (40.91), N (2914), ^4 ^= df (4/2909), F (12.67), N (2914)

PtSS: Posttraumatic stress symptoms

SD: standard deviation

G1 = Were injured by the Tsunami, G2 = Witnessed the Tsunami, G3 = Injured or dead family members, G4 = Injured or dead friends/acquaintance, lost property, G = 5 No exposure

PH: Post-hoc

**Figure 1:**
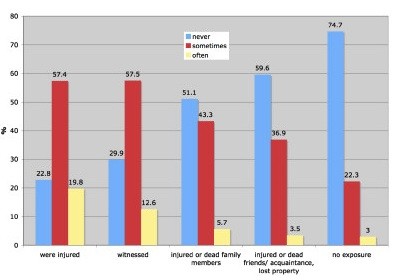
**Frequency of nightmares and flashbacks in the five sub-samples grouped by exposure severity**. Blue bars indicate that only one in four respondents who were injured had no flashbacks and nightmares later. The proportion of those who reported occasional (red) or frequent (yellow bars) flashbacks and nightmares declines according to the severity of the exposure. Note that the differences between those who were actually injured and those who "only" witnessed is small.

These results show that higher scores in the traumatic stress and depression scales could be identified in those directly affected by the incident. However, obsessive-compulsive symptoms did not differ within these groups. In order to better understand the association between self reported mental health symptoms and various degrees of exposure to the disaster, the prevalence of specific key symptoms was analyzed. These analyses revealed that subjects who were injured by the Tsunami displayed the highest prevalence rate of nightmares and flashbacks: One out of five victims (19.8%, N = 32) who witnessed the Tsunami waves reported frequent nightmares and flashbacks of the incident. In the group of those subjects who were not directly affected by the Tsunami, only 3.0% (N = 51) reported a frequent re-experience of the disaster. Figure 1 shows the frequency of reported nightmares and flashbacks in the five sub-samples grouped by exposure severity.

**Figure 2:**
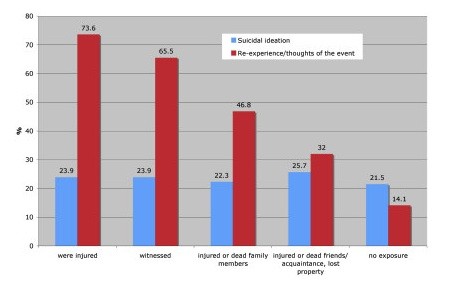
**Frequency of suicidal ideation and re-experience/thoughts of the event in the five sub-samples grouped by exposure severity**. Red bars indicate that three out of four respondents who were injured suffered from re-experiencing the catastrophe. This prevalence rate declines linearly across the exposure groups reaching its lowest for those who were not directly affected. Blue bars indicate that roughly one out of four ONSET users reported suicidal ideation - irrespective of the exposure.

As shown in Figure 2, the prevalence of suicidal ideation varies across the five strata between 21.5% and 25.7% suggesting that the exposure strata did not differ substantially with respect to suicidal ideation. Accordingly, the bivariate regression analysis, using suicidal ideation as the dependent and the severity of exposure (group) as the independent variable, did not reveal any significant differences (see Table 6). However, the five groups showed significant differences with respect to the prevalence of re-experience of the event. Whereas nearly three out of four users injured by the Tsunami reported some degree of re-experience (73.6%, N = 120), in the group of those subjects not directly affected, less than one fifth (14.7%, N = 249) reported suffering from some level of re-experience.

**Table 6:** Bivariate regression analysis, using Group 1 as the reference group in the dummy variable

		OR	P	95% CI
Suicidal ideation Reference group: Group 1	Group 1 versus Group 2	0.9	0.68	0.6-1.4
	
	Group 1 versus Group 3	1.0	0.92	0.6-1.7
	
	Group 1 versus Group 4	1.1	0.66	0.7-1.7
	
	Group 1 versus Group 5	0.9	0.47	0.6-1.3

Re-experience/Thoughts of the event Reference group: Group 1	Group 1 versus Group 2	0.7	0.06	0.5-1.0
	
	Group 1 versus Group 3	0.3	0.00	0.2-0.5
	
	Group 1 versus Group 4	0.2	0.00	0.1-0.3
	
	Group 1 versus Group 5	0.1	0.00	0.0-0.1

OR: odds ratio

CI: confidence interval

## Discussion

According to the British NICE clinical guideline, it is recommended to routinely use a brief screening instrument for PTSD one month after a disaster [21]. After natural disasters, the routine screening of the affected population - using e.g. telephone monitoring - is very cost-intensive. Besides the financial aspect, in order to reach out to the victims, the authorities would have to know who was affected. A pragmatic and cost-efficient alternative to telephone or direct, face-to-face monitoring is the use of Internet-based questionnaires.

The objective of this article was to report the insights that were gained using an Internet-based self-screening instrument after a large-scale natural disaster. The development of the instrument was the result of a crisis-assessment of the Swiss Federal Office for Civil Protection that concluded that there were not enough trained professionals available who could personally contact all persons who claimed to be affected by the disaster. In January 2005 the Swiss authorities were not aware of the existence of an already published multi-lingual trauma related online self-evaluation instrument and decided thus to engage in the development of such an instrument. It is, however, important to stress that this course of action inevitably led to a series of severe shortcomings. These become more evident when ONSET is compared to another online self-evaluation instrument that was implemented in the aftermath of Hurricane Katrina. The authors relied on well-validated instruments and were able to assess socio-demographic information as well as important information regarding trauma-relevant preconditions. For ONSET - besides the PTSS - due to copyright issues and the difficulty of the multi-lingual design of the instrument, no validated instruments could be used and thus it was not possible to compare the ONSET users to a norm population. Since the developers of ONSET did not have the time to commission a legal expert opinion regarding data protection of a web-based mental health instrument, it was decided to guarantee very strict anonymity to users. This decision led to a severe limitation in interpreting the data, since no pre-existing traumatic events were assessed. Theses serious difficulties lead to a first, not very surprising conclusion, namely to develop an online instrument before a catastrophe.

Despite these shortcomings, the developers were surprised by the large number of respondents: ONSET was available for a period of six and a half months and was filled out by 4,161 adult users, providing 2,914 datasets of persons who gave their informed consent for subsequent statistical analyses. Though it is not possible to quantify the outreach in terms of proportion (how many of the victims were reached?), the absolute numbers give an indication of the demand of such an instrument. The launch of ONSET was accompanied by a rather impressive media campaign, was mentioned in all nationally available newspaper, on radio, and also on national TV. Furthermore it was advertized on the website of the Swiss Ministry of Foreign Affairs. The developers were thus interested to find out, whether this campaign paid out in the sense that ONSET was primarily used by the population that was directly informed about the instrument. The descriptive analyses reveal that more than four out of five respondents (81.1%) reported to be Swiss residents or Swiss nationals. Though not corroborated using a control group design, this result led to a second conclusion, namely that the launch of a web-based instrument should be accompanied by a public relation campaign to ensure a substantial outreach. A further indication of the outreach of ONSET can be documented by the finding that a total of 467 Swiss users claimed to have been directly affected by the Tsunami wave. Although the exact figures of the number of affected Swiss persons was not known, several reports indicated that 2'000 to 3'000 Swiss tourists were affected by the disaster and thus a considerable proportion of Swiss who were affected used ONSET.

When assessing the outreach of the instrument, another central question is, whether those respondents who used the instrument, obtained high scores in the trauma-related scales. Among the 2'914 users, the mean score of the PTSS-10 was 12 points. Using a cut-off of 12 points, 45.04% of the users reported a degree of symptoms relevant to PTSD. The highest mean score of the PTSS could be found in those users who claimed to have personally witnessed the Tsunami (14.6%) followed by those users who were grieving over the loss of friends or family (13.4%). The prevalence of 45% of the users reporting PTSD relevant symptoms differs substantially from epidemiological studies, which had reported a PTSD prevalence of 4.5% after a large-scale disaster [8] and is at the upper bound of the 5-60% interval presented by Galea et al. [1]. However, results gathered from ONSET users after the Tsunami-wave are not conclusive in the sense that they do not give a robust estimate of the PTSD prevalence in the Swiss citizen affected by the Tsunami since we don't have reliable information of the ONSET outreach. Besides the general difficulties and uncertainties that accompany the implementation of a new screening strategy, ONSET was developed as a cross-sectional instrument. Carrying out a screening just at one appointed date might fail to reach all affected individuals [22].

ONSET was primarily used within the first month after its launch. Unlike other research we did not find important differences between time of use of the instrument and type of exposure. Covell et al. [23] for example reported that victims of the terrorist attacks of the World Trade Centre in the U.S. who lost family members were mainly accessing mental health services during the first month of availability while victims being affected by displacement or loss of employment were seeking support evenly within the observed period of time. Having a closer look on the two projects investigated we assume that due to different services provided by Project Liberty (short-term counseling and intervention) and ONSET (pure screening instrument including the advice to seek intervention) might have led to different results. Whether pure screening reaches out to a larger proportion of the affected population than counseling services cannot be answered so far, though it seems to be plausible.

When analyzing the gender composition of ONSET users it becomes evident, that web-based instruments are to some extent gender biased: 57.5% of the users were male and 42.5% were female. This finding is in line with other research results showing that males use the Internet more frequently and for other purposes than females [24]. They express more positive attitudes than did females on two aspects of the Internet: usefulness and perceived control [25]. Since research has shown, that men usually less often than women seek treatment for psychiatric disorders ONSET makes a great effort in reaching male victims [26]. Whether ONSET helped to reach out to a group of victims who was traditionally reluctant in seeking treatment or whether ONSET was not presented in a way that was equally attractive to women, cannot be answered.

### Limitations

In our study, not only participants directly affected by the Tsunami reported a relatively high score on the trauma scale, but also persons who were not directly affected displayed an elevated score on the trauma scale. This finding could be explained by potential preexisting trauma of some ONSET respondents. Since ONSET did not assess any prior traumatizing events, it is not possible to control the analyses for other trauma than those inflicted by the Tsunami. To get a better understanding of that group not being directly affected by a disaster but still searching for support more detailed data about the motivation leading to participation in a PTSD-screenings should be recorded in further research. Furthermore, the data is cross-sectional and for this reason it remains impossible to determine whether the self reported mental health problems are the result of the Tsunami, or rather whether they represent the exacerbation of pre-existing problems. Some users sent us email testimonials of earlier trauma reactivation due to the Tsunami media coverage.

No data about the utilization of mental health service before the disaster were collected. Since this variable has been shown to mediate utilization of mental health services after the disaster it should be included in further research to be able to more clearly interpret the effect of Internet-based screenings on seeking mental health services after the disaster.

Another concern might be that the study population is a convenience sample even if it is of considerable size and it is thus not possible to assess to what extent, and with regard to which characteristics, the sample is biased (when compared to the population of all Tsunami victims). Furthermore, in order to use ONSET, knowledge of its existence had to be known. Potential users had to have access to the Internet and basic computer skills, which explains some of the highly homogenous socio-demographic characteristics of the study sample.

A further limitation is that no objective information was available beside the self-reports. From the beginning data were assessed strictly anonymously. Whether this might even increase the validity of the data in this context cannot be answered. However, when analyzing the descriptive results, there were virtually no striking outliers, such as unusual declarations of old age that could have been interpreted as voluntary or involuntary false and misleading statements. Our results coincide with findings of other studies in the field, but nonetheless the comparability is limited since the period of time that ONSET was available differs from requirements of the NICE-guideline and with that from most procedures in other studies. Furthermore, with the exception of very few odd answering patterns that led to the exclusion of those subjects from the analyses, we did not identify striking response behavior that could be interpreted as a limitation of the internal validity of ONSET. We therefore assume that there is a certain degree of empirical evidence which supports the overall validity of the study data, and hence support the use of online assessment instruments for public mental health monitoring. As a side finding, our ONSET sub-versions for children and adolescents of less than 17 years old were used only 37 times. It indicates a lower value of an Internet-based screening tool for children and adolescents who have experienced a critical incident.

A last limitation we see is the concern if given recommendations of the online tool are followed-up on by participants. A conclusion about respondents actually effectively seeking or not seeking mental health treatment cannot be drawn from this study. It is also debatable whether advice obtained digitally and online is taken as seriously as advice being given in a face-to-face conversation [27]. We recommend further research on this issue, as it is vital to determining the usefulness of Internet-based screening instruments.

## Conclusions

We interpret the findings as a first indicator that online-screenings after large scales disasters are a valuable addendum to conventional forms of screenings. ONSET helped reaching out to individuals who were not directly affected by the Tsunami, but showed nevertheless a high level of traumatization. This instrument helped a significant proportion of subjects who thought to be affected by the catastrophe to decide, whether they wanted to see a mental health professional. Furthermore, men more frequently accessed the instrument than women, indicating that Internet-based testing facilitates reaching out to a different group of people than "ordinary" public mental health strategies.

Therefore ideally, ONSET or similar online screenings should be one element of adequate mental health care being provided for all affected individuals following major traumatic events.

## Competing interests

The authors declare that they have no competing interests.

## Authors' contributions

SV developed ONSET and carried out the study design and was involved in writing the manuscript and has given final approval of the version to be published. JE has given substantial contributions to conception, to analysis, interpretation of the data and helped to draft the manuscript. He has given final approval of the version to be published. TE, AL, FU, JG and WR have been involved in revising the manuscript critically and have given final approval of the version to be published. AR performed the statistical analyses, was involved in interpreting of the results and writing the manuscript. All authors read and an approved the final manuscript.

## Pre-publication history

The pre-publication history for this paper can be accessed here:

http://www.biomedcentral.com/1471-2458/11/18/prepub
